# Clinical significance of the loss of CD20 antigen on tumor cells in patients with relapsed or refractory follicular lymphoma

**DOI:** 10.20517/cdr.2020.109

**Published:** 2021-03-26

**Authors:** Jean-Marie Michot, Alice Buet-Elfassy, Maxime Annereau, Julien Lazarovici, Alina Danu, Clémentine Sarkozy, Claude Chahine, Camille Bigenwald, Jacques Bosq, Julien Rossignol, Patricia Romano-Martin, Capucine Baldini, David Ghez, Peggy Dartigues, Christophe Massard, Vincent Ribrag

**Affiliations:** ^1^Gustave Roussy, Université Paris-Saclay, Département des Innovations Thérapeutiques et Essais Précoces, Villejuif 94800, France.; ^2^Gustave Roussy, Université Paris-Saclay, Pharmacy Departement, Villejuif 94800, France.; ^3^Gustave Roussy, Université Paris-Saclay, Departement of Hematology, Villejuif 94800, France.; ^4^Gustave Roussy, Université Paris-Saclay, Departement of Pathology, Villejuif 94800, France.

**Keywords:** Follicular lymphoma, CD20 tumor antigen, anti-CD20 monoclonal antibody, cancer drug resistance

## Abstract

**Aim:** Anti-CD20 monoclonal antibody is a cornerstone therapy for follicular lymphoma. Following anti-CD20 therapy, a potential decrease in CD20 antigen, and therefore a loss of the tumor target might be expected. However, the incidence and clinical significance of CD20 loss on tumor cells in patients with relapsed or refractory follicular lymphoma are unknown. This study aims to investigate the incidence and outcome of patients with relapsed or refractory follicular lymphoma patients harboring the loss of the tumor target, CD20.

**Methods:** All consecutive adult patients with relapsed or refractory follicular lymphoma referred to the Early Drug Department at Gustave Roussy were included. The main objectives were to assess the incidence and prognosis of the loss in expression of CD20 antigen on the surface of tumor cells on patient outcome.

**Results:** Over the study period 2013-2018, 131 patients were screened for clinical trials with B-cell malignancies in the early drug department of Gustave Roussy in France. Forty-four patients presented with relapsed or refractory follicular lymphoma and 32 had tumor biopsies at the time of relapse that were retained for analysis. The median (range) age was 67.5 years (55.3-75.3) and the median number of prior anti-cancer systemic therapies was 3 (2-4). At the time of relapse, CD20 expression was positive in 84% of tumors (*n* = 27) and negative in 16% of tumors (*n* = 5). At a median follow-up of 18.3 (0.6-83.3) months, CD20 negativity was associated with a poorer prognosis with a median overall survival of 8.9 months (95%CI: 2.4-19.1) in comparison to CD20 positive patients (28.3 months, 95%CI: 25.1-75.3 months, *P* = 0.019).

**Conclusion:** The loss of the tumor target antigen, CD20, occurred in 16% of patients with relapse or refractory follicular lymphoma. Due to confounding factors in patients who received anti-CD20 immunotherapy, it was not possible to formally establish the prognostic significance of CD20 negativity. However, we suggest that a check for CD20 antigen positivity nevertheless be performed to adapt subsequent therapies for patients with relapsed or refractory follicular lymphoma.

## INTRODUCTION

Rituximab, the first generation of therapeutic anti-CD20 monoclonal antibody, is approved alone or in combination with chemotherapy as the standard care of follicular lymphoma^[1]^. Rituximab combined with lenalidomide recently demonstrated meaningful activity against follicular lymphoma as a first-line of therapy^[2]^. Rituximab is also recommended as a first-line of maintenance therapy for follicular lymphoma^[3]^. Compared with rituximab, the second generation anti-CD20 monoclonal antibody, obinutuzumab, combined with chemotherapy improved treatment efficacy in patients with follicular lymphoma^[4]^. As with most targeted therapies, a potential mechanism that tumor cells employ to evade treatment is the modulation of the putative target, in the case of follicular lymphoma, the expression of CD20. Several mechanisms of actions of the therapeutic drug have been proposed including CD20 down-regulation, modification of CD20 glycosylation, as well as CD20 protein folding and epigenetic changes^[5]^. Internalization of the CD20 protein in the cytoplasm of tumor cells and Fcγ receptor polymorphisms have also been suggested to offer resistance against anti-CD20 antibody^[6,7]^.

Although studies have reported previously that patients with B-cell follicular lymphoma show a loss of expression of the CD20 antigen on tumor cells^[8-11]^, the incidence of this loss and its potential impact on patient’s outcome remains unknown. This study aims to investigate the clinical significance of the loss of expression of CD20 antigen on tumor cells in patients with relapsed or refractory follicular lymphoma.

## METHODS

### Patients

**Figure 1:**
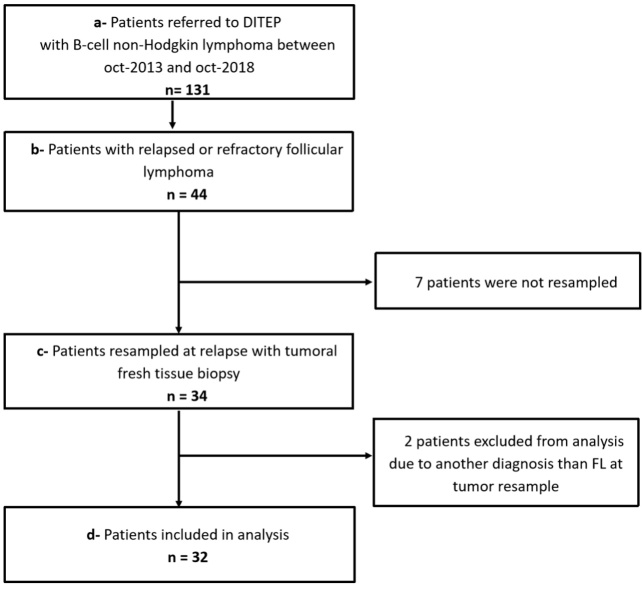
Flow chart of patients included in the study. DITEP: Département des Innovations thérapeutiques et essais précoces.

This study included consecutive patients aged over 18 years with relapsed or refractory follicular lymphoma referred to the *Département des Innovations Thérapeutiques et Essais Précoces* of Gustave Roussy, Villejuif, France during the period October 2013-October 2018. Patients retained for analysis were required to have biopsy-proven relapsed or refractory follicular lymphoma including immunohistochemical (IHC) CD20 staining. All tumor histological specimens were fixed in formaldehyde-acetic acid-alcohol and stained with hematoxylin-eosin-safran and Giemsa. Immunohistochemistry of tumor tissue included the staining for CD20, CD79a, CD19, CD10, BCL2, CD5, CD23 antigens. A central pathology review was conducted by 2 independent hematopathologists (PD and JB) according to the 2016 WHO classification^[12]^ and follicular lymphoma (FL) grading was assessed at the time of tumor re-biopsy. All patients had a complete physical examination, viral serologies including hepatitis B, hepatitis C and Human Immunodeficiency Virus testing, and computerized tomography body scan, according to institutional guidelines. The Institutional Review Board and local Ethic Committee of Villejuif approved the study. All patients gave their written informed consent for the study. All the patients signed a written consent for the use for research purposes of the remnants of samples taken for diagnostic and/or therapeutic purposes with the Institutional Review Board and local Ethics Committee agreement number L1211-2. Between October 2013 and October 2018, 131 patients with relapsed or refractory B-cell non-Hodgkin lymphoma were referred to the *Département des Innovation Therapeutiques et Essais Précoces* of Gustave Roussy, Villejuif, in France. Among them, 44 patients were referred with a relapsed or refractory follicular lymphoma and 34 of them had been rebiopsied. Two patients were excluded from analysis after central pathological review (one patient with lung carcinoma and one patient with marginal zone lymphoma) [Figure 1], resulting therefore in 32 patients with relapsed or refractory follicular lymphoma for final analysis.

### Clinical characteristics

The clinical characteristics were collected from the patient's medical records, and included age, gender, Ann Arbor staging at diagnosis and relapse, number of previous lines of systemic therapy before tumor resample, and outcome data. The characteristics of previous anti-CD20 therapies were collected including time from the last anti-CD20 infusion, tumor resamples, and the cumulative number of anti-CD20 infusions before tumor resample. All clinical and laboratory data tables are provided in Supplementary Table 1.

### Monoclonal antibodies anti-CD20

The anti-CD20 monoclonal antibodies that were considered in the study were rituximab intravenously and obinutuzumab intravenously, both supplied by Roche. The treatments were given to patients under the marketing authorization and were administered according to institutional recommendations based on standard doses in routine care.

### Statistical analyses

Based on the CD20 immunochemistry analysis at tumor resample, patients were classified as CD20 positive or CD20 negative. The CD20 negativity was retained when 0% of CD20 positive tumor cells were observed by microscope analysis. The period of follow-up was defined as the time between tumor resample and last follow-up or death from any cause. Overall survival (OS) was defined as the time from tumor resample until death from any cause or last follow-up. Qualitative variables were reported as the number and frequency, and quantitative variables were reported as the median with the standard deviation (SD). Patients group comparisons were performed with the Fisher’s exact test for qualitative variables, a *t*-test for quantitative variables, or the Wilcoxon’s test if data were not normally distributed. A *P*-value of < 0.05 was considered statistically significant. Survival was estimated with the Kaplan Meier method on SAS 9.3 software (SAS Institute, Cary, NC, USA). Data were quoted as the median (range).

## RESULTS

### Patient characteristics

The clinical features of 32 patients retained for analysis are shown in Table 1. Median age was 67.5 years (IQR: 55.3-75.3 years) at the time of tumor resampled biopsy. At relapse, all patients had a lymph node biopsy, except one patient who had a bone marrow biopsy for bone marrow involvement of follicular lymphoma. All patients were previously treated with rituximab combined with anthracycline-based chemotherapy as first-line therapy, followed by rituximab maintenance. The median of previous lines of systemic therapies was 3 (IQR: 2-4) and 16 (50%) patients had received previous auto-stem cell transplantation.

**Table 1:** Clinical features of patients with relapsed or refractory CD20-positive and CD20-negative follicular lymphoma follicular

**Patient’s clinical features (at relapse or refractory timepoint)**	**Total R/R FL patients** **(*n* = 32 pts)**	**R/R FL patients CD20 positive** **(*n* = 27 pts)**	**R/R FL patients CD20 negative** **(*n* = 5 pts) **	***P*** **-value**
Age, median (IQR) (years)	67.5 (55.3-75.3)	68.1 (54.1-75.3)	64.3 (64.4-75.2)	*P* = 0.876
Gender (%)				
Male	17 (53)	15 (56)	2 (40)	*P* = 0.645
Female	15 (47)	12 (44)	3 (60)	
Ann Arbor stage (%)				
Localized I-II	2 (6)	2 (7)	0	
Extended III-IV	30 (94)	25 (93)	5 (100)	*P* = 0.160
Time from diagnosis to biopsy, median (IQR) (months)	74.1 (45.3-128.8)	82.2 (49.0-131.5)	46.3 (35.1-73.3)	*P* = 0.275
Number of lines of therapy before sample, median (IQR)	3 (2-4)	3 (2-4)	4 (4-5)	*P* = 0.079
Number of anti-CD20 infusions before sample, median (IQR)	20 (12-25)	20 (13-24)	25 (12-30)	*P* = 0.515
Time from last anti-CD20 infusions before sample, median (IQR) (months)	11.1 (4.8-21.3)	11.5 (7.1-25.3)	2.1 (1.0-5.4)	*P* = 0.011
Obinutuzumab pre-exposition (%)				
Yes	1 (3)	0	1 (20)	
No	31 (97)	27 (100)	4 (80)	*P* = 0.505
Auto stem-cell transplant (%)				
Yes	16 (50)	14 (52)	2 (40)	
No	16 (50)	13 (48)	3 (60)	*P* = 0.510
WHO classification* (%)				
Grade 3b	10 (31)	9 (33)	1 (20)	
Other grades	22 (69)	18 (67)	4 (80)	*P* = 0.234
Time from diagnosis to last news, median (IQR) (months)	93.3 (63.1-154.2)	103.0 (68.5-158.3)	62.1 (50.2-85.4)	*P* = 0.126
Time from resample to last news, median (IQR) (months)	19.1 (8.3-26.4)	19.3 (9.8-27.5)	8.9 (4.5-11.5)	*P* = 0.076

^*^According to the WHO 2016 classification. R/R FL: Relapsed or refractory follicular lymphoma; IQR: interquartile; Pts: patients; WHO: World Health Organization.

### Clinical features associated with CD20 loss on tumor cells

**Figure 2:**
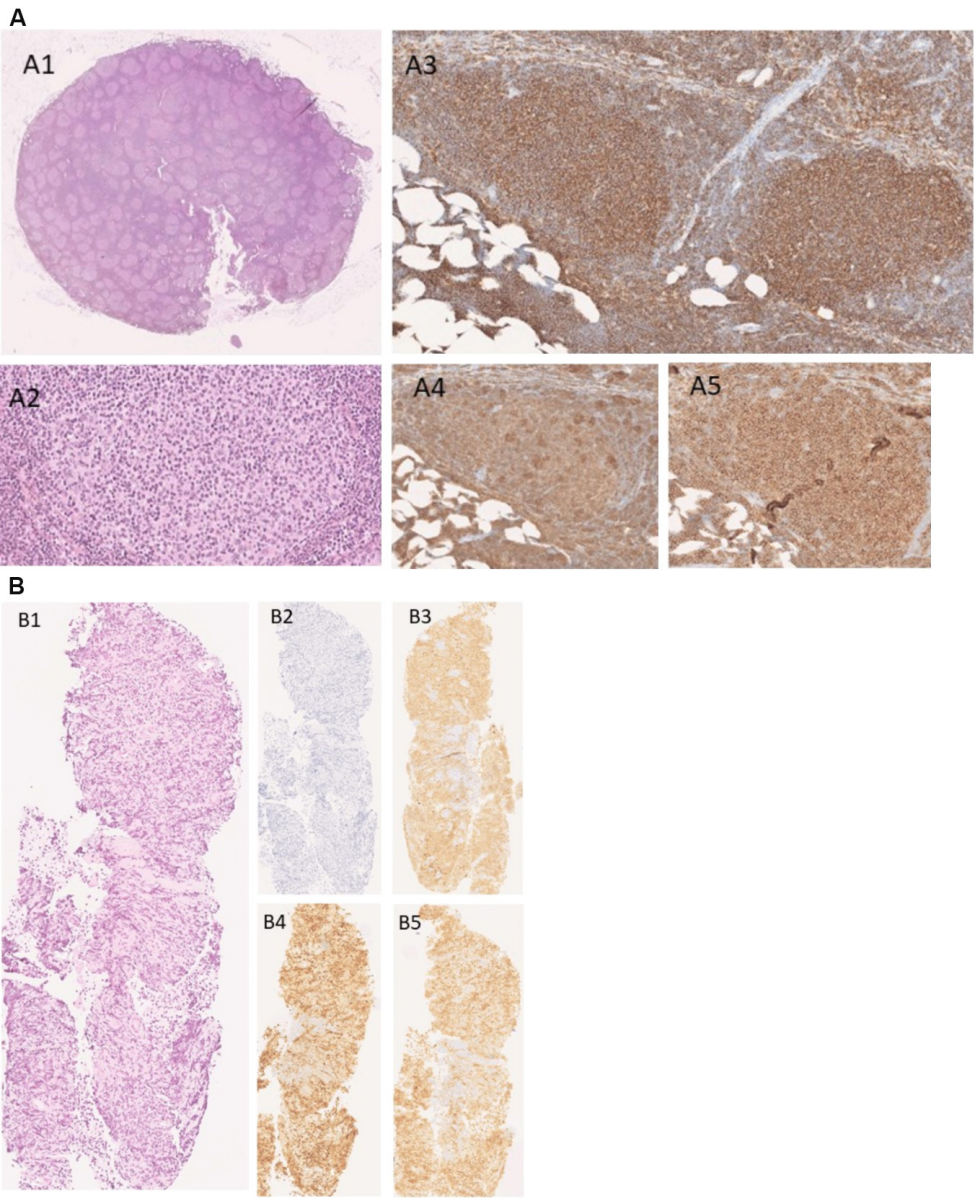
Patients with follicular lymphoma at the time of diagnosis were CD20 positive (A). Neoplastic follicles replaces nodal architecture with back-to-back pattern (HES X 1) (A1). Low grade (1-2) with a predominance of centrocytes (HES X20) (A2). CD20 positive B cells are seen in both follicular and interfollicular areas (HES X 20) (A3). Staining CD10 (A4) and BCL2 (A5) were found positive within the follicles and also infiltrate interfollicular areas (HES X20). Follicular lymphoma (X10) of the same patient at relapse and after anti-CD20 therapy (rituximab) with loss of CD20 (B). Neoplastic follicles in low-grade follicular lymphoma, low grade (grade 1-2) with predominance of centrocytes (B1). Neoplastic follicles replaces nodal architecture with back-to-back pattern (HES X 10). Immunohistochemistry with immunoperoxidase stains in paraffin sections shows CD20 negative B cells (B2). The other B cell markers CD79A (B3), PAX5 (B4), and BCL6 (B5) remain positively expressed by follicular lymphoma cells.

Among the 32 patients included in the study, the loss of CD20 expression on tumor cells was observed in 5 patients (16%). The tumor biopsies of the 5 diagnosed patients with CD20 loss were centrally reviewed (by pathologists PD and JB) and the initial diagnosis of CD20 positive follicular lymphoma was also confirmed for all 5 patients. An example of a follicular lymphoma biopsy sample from a patient initially diagnosed with CD20 positive lymphoma who lost CD20 at relapse after anti-CD20 treatment (rituximab) is shown in Figure 2. The age, sex ratio, number of previous numbers of systemic therapies, and autologous stem-cell transplantation were similar in the CD20 positive and CD20 negative patient groups [Table 1]. The median time between the last infusion of anti-CD20 antibody and tumor resample was found to be shorter in the CD20-negative group (2.1 months, IQR: 1.0-5.4) compared with the CD20-positive group (11.5 months, IQR: 7.1-25.3, *P* = 0.012). In the 5 patients with CD20 negative follicular lymphoma at tumor resample, the expression of other B-cells markers of FL by immunochemistry (CD79a and CD19) was assessed. CD79a remained positive in 4 out of the 4 patients tested and CD19 remained positive in 3 out of the 3 patients tested for these parameters [Table 2].

**Table 2:** Expression of B-cells markers in tumor cell surfaces (CD20, CD79a, CD10, CD19) in the CD20 negative patients with relapsed or refractory follicular lymphoma at resample time on fresh tumor biopsy. All tests were performed by immunohistochemistry

	**CD20**	**CD79a**	**CD10**	**CD19**
**Pt #15**	-	+	+	NA^*^
**Pt #22**	-	+	+	+
**Pt #24**	-	+	+	+
**Pt #26**	-	NA^*^	+	NA^*^
**Pt #28**	-	+	+	+

^*^The tumor material was sold out and not sufficient to perform these analyses. NA: Not available.

### The outcome of patients with CD20 loss in tumors

**Figure 3:**
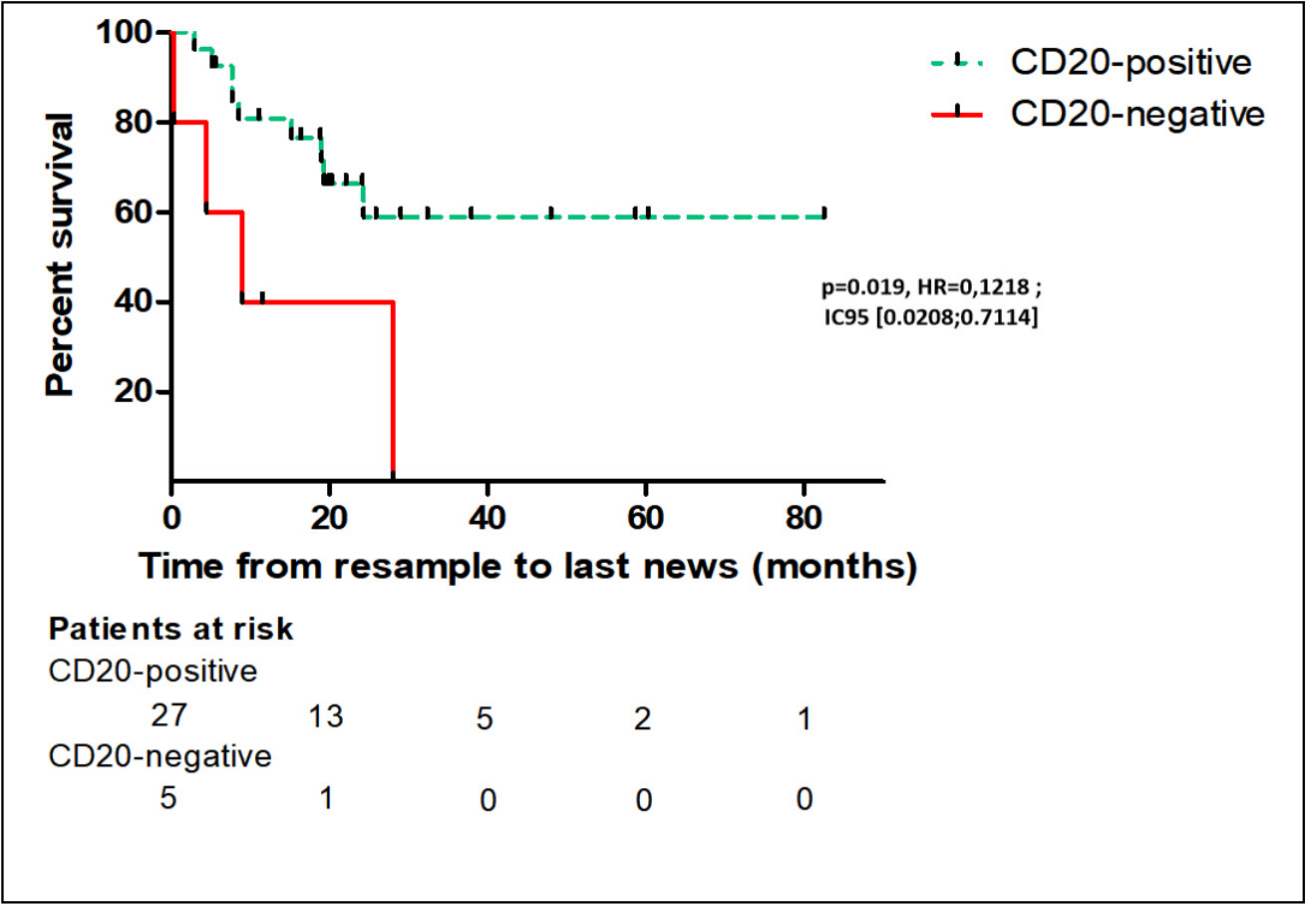
Overall survival of patients with relapsed or refractory follicular lymphoma, according to the CD20 positive or negative status at tumor resample. Comparison of overall survival was done by performing a log-rank test. A significant difference in overall survival between the groups was found (*P* = 0.019; HR = 0,1218; IC95 [0.0208;0.7114]).

With a median follow-up of 18.3 (0.6-83.3) months, the estimated median OS in the CD20-positive patient group was 28.3 months (95%CI: 25.1-75.3 months). The median OS in the CD20-negative patient group was 8.9 months (95%CI: 2.4-19.1). The differences were statistically significant (*P* = 0.019) [Figure 3].

### DISCUSSION

The loss of CD20 expression at tumor relapse was observed in 16% of patients with relapsed or refractory follicular lymphoma in our study. This loss was associated with a poorer outcome when compared with follicular lymphoma patients that were CD20 positive.

The occurence of CD20 negativity in patients with follicular lymphoma at the follow-up is relatively consistent with results from patients with non-Hodgkin lymphoma, ranging from 12% of patients in Foran’s study^[13]^ and 26% of patients in Hiraga’s study^[5]^. Our study revealed that CD20 negativity was associated with a poorer outcome. Detailed investigation of our data showed that the time from the last infusion of anti-CD20 antibody to tumor resample was shorter in the CD20 negative patient group. This result suggests that the loss of CD20 tumor antigen expression might be a transient down-regulation phenomenon induced by anti-CD20 therapy, as suggested by other studies^[5,13,14]^. Multiple mechanisms can cause loss of CD20 expression in lymphoma and acquisition of rituximab resistance could be associated with deregulations at both the pretranscriptional and posttranscriptional levels^[15]^. Some studies found that mutations of the *MS4A1* gene could be linked at least partially with the loss of CD20 antigen^[15,16]^. Recent studies suggest the predominance of post-transcriptional epigenetic changes of the CD20 antigen protein folding involved in the loss of CD20^[5,17]^.

In our study, patients with the CD20 negative tumors remained positive for other B-cells markers such as CD19 or CD79a. These data suggest that treatment with an anti-CD20 monoclonal antibody remains specific to CD20 antigen downregulation and spares the other B cells antigens. Therefore, checking for tumor antigen positivity seems important in light of the recent development of new antigen-specific immunotherapies in non-Hodgkin lymphoma. Recent therapies that were approved in the year 2020 targeted CD19 antigen such as CD19 CAR-T cells^[18]^, as well as recent therapies against the CD79 antigens such as polatuzumab antibody drug conjugates^[19]^. Furthermore, there is currently a significant development of bi-specific T-cell engaging CD3xCD19 and CD3xCD20 in clinical trials^[20]^, as well as of a combination of CD20 antibodies with oral immunomodulatory drugs^[21,22]^. In this context of multiple new possibilities of modern immunotherapies that targetd B-cell receptors by, a verification of the tumor antigen target before the initiation of treatment isa viable approach.

The main limitations of this study are the limited size of the sample of patients with CD20 negativity, and its retrospective design. We also acknowledge a potential selection bias related to the tertiary center nature of our site, selecting the most “hard-to-treat” patients with relapsed or refractory lymphomas. In our study, few patients with CD20 negative tumors were identified, and we acknowledge that more cases identified in a larger cohort of patients would add power to the analysis. The limited size of patients in the with CD20 negative group do not allow for any definitive conclusions, and further studies are warranted to investigate if CD20 loss on tumor cells is indeed associated with poorer outcomes in patients with relapse or refractory follicular lymphoma. We recognize that it was not possible to formally establish the prognostic significance of CD20 negativity in patients who received anti-CD20 immunotherapies. Indeed, a major confounding factor was that the poor prognosis of this subgroup of patients may be linked more to an early relapse than to CD20 negativity.

The loss of the tumor target antigen, CD20, occurred in 16% of patients with relapse or refractory follicular lymphoma. Due to confounding factors in patients who received anti-CD20 immunotherapy, it was not possible to formally establish the prognostic significance of CD20 negativity. However, we suggest that a check for CD20 antigen positivity nevertheless be performed to adapt subsequent therapies for patients with relapsed or refractory follicular lymphoma, especially when immunotherapy against tumor target CD20 is targeted.

## DECLARATIONS

### Availability of data and materials

The authors indicate that their data with raw data could be found in Supplementary Table 1 in the supplementary material.

### Copyright

© The Author(s) 2021.

## References

[1] Molina A (2008). A decade of Rituximab: Improving survival outcomes in non-Hodgkin’s lymphoma. Annu Rev Med.

[2] Morschhauser F, Fowler NH, Feugier P (2018). Rituximab plus Lenalidomide in advanced untreated follicular lymphoma. N Engl J Med.

[3] Salles G, Seymour JF, Offner F (2011). Rituximab maintenance for 2 years in patients with high tumour burden follicular lymphoma responding to rituximab plus chemotherapy (PRIMA): a phase 3, randomised controlled trial. Lancet.

[4] Singh V, Gupta D, Almasan A (2015). Development of novel anti-Cd20 monoclonal antibodies and modulation in Cd20 levels on cell surface: Looking to improve immunotherapy response. J Cancer Sci Ther.

[5] Hiraga J, Tomita A, Sugimoto T (2009). Down-regulation of CD20 expression in B-cell lymphoma cells after treatment with rituximab-containing combination chemotherapies: its prevalence and clinical significance. Blood.

[6] Jilani I (2003). Transient down-modulation of CD20 by rituximab in patients with chronic lymphocytic leukemia. Blood.

[7] Cartron G, Dacheux L, Salles G (2002). Therapeutic activity of humanized anti-CD20 monoclonal antibody and polymorphism in IgG Fc receptor FcgammaRIIIa gene. Blood.

[8] Haidar JH, Shamseddine A, Salem Z (2003). Loss of CD20 expression in relapsed lymphomas after rituximab therapy. Eur J Haematol.

[9] Bagacean C, Quintin-Roue I, Ianotto JC (2015). CD20 negative relapse of a follicular lymphoma after chemoimmunotherapy. HVM Bioflux.

[10] Álvaro-Naranjo T, Jaén-Martínez J, Gumá-Padró J (2003). CD20-negative DLBCL transformation after rituximab treatment in follicular lymphoma: a new case report and review of the literature. Ann Hematol.

[11] Davis TA, Czerwinski DK, Levy R (1999). Therapy of B-cell lymphoma with anti-CD20 antibodies can result in the loss of CD20 antigen expression. Clin Cancer Res.

[12] Swerdlow SH, Campo E, Pileri SA (2016). The 2016 revision of the World Health Organization classification of lymphoid neoplasms. Blood.

[13] Foran JM, Norton AJ, Micallef INM (2001). Loss of CD20 expression following treatment with rituximab (chimaeric monoclonal anti-CD20): a retrospective cohort analysis. Br J Haematol.

[14] Maeshima A, Taniguchi H, Fukuhara S (2013). Follow-up data of 10 patients with B-cell non-Hodgkin lymphoma with a CD20-negative phenotypic change after rituximab-containing therapy. Am J Surg Pathol.

[15] Czuczman MS, Olejniczak S, Gowda A (2008). Acquirement of rituximab resistance in lymphoma cell lines is associated with both global CD20 gene and protein down-regulation regulated at the pretranscriptional and posttranscriptional levels. Clin Cancer Res.

[16] Johnson NA, Leach S, Woolcock B (2009). CD20 mutations involving the rituximab epitope are rare in diffuse large B-cell lymphomas and are not a significant cause of R-CHOP failure. Haematologica.

[17] Tsutsumi Y, Ohigashi H, Ito S (2016). 5-Azacytidine partially restores CD20 expression in follicular lymphoma that lost CD20 expression after rituximab treatment: a case report. J Med Case Reports.

[18] Chow VA, Shadman M, Gopal AK (2018). Translating anti-CD19 CAR T-cell therapy into clinical practice for relapsed/refractory diffuse large B-cell lymphoma. Blood.

[19] Morschhauser F, Flinn IW, Advani R (2019). Polatuzumab vedotin or pinatuzumab vedotin plus rituximab in patients with relapsed or refractory non-Hodgkin lymphoma: final results from a phase 2 randomised study (ROMULUS). Lancet Haematol.

[20] Bukhari A, Lee ST (2019). Blinatumomab: a novel therapy for the treatment of non-Hodgkin’s lymphoma. Expert Rev Hematol.

[21] Hiddemann W, Barbui AM, Canales MA (2018). Immunochemotherapy with Obinutuzumab or Rituximab for previously untreated follicular lymphoma in the GALLIUM study: influence of chemotherapy on efficacy and safety. J Clin Oncol.

[22] Morschhauser F, Le Gouill S, Feugier P (2019). Obinutuzumab combined with lenalidomide for relapsed or refractory follicular B-cell lymphoma (GALEN): a multicentre, single-arm, phase 2 study. Lancet Haematol.

